# Pilot Study on the Use of Untargeted Metabolomic Fingerprinting of Liquid-Cytology Fluids as a Diagnostic Tool of Malignancy for Thyroid Nodules

**DOI:** 10.3390/metabo13070782

**Published:** 2023-06-23

**Authors:** Grégoire D’Andréa, Lun Jing, Isabelle Peyrottes, Jean-Marie Guigonis, Fanny Graslin, Sabine Lindenthal, Julie Sanglier, Isabel Gimenez, Juliette Haudebourg, Clair Vandersteen, Alexandre Bozec, Nicolas Guevara, Thierry Pourcher

**Affiliations:** 1 Otorhinolaryngology and Head and Neck Surgery Department, Institut Universitaire de la Face et du Cou, GCS Nice University Hospital—Antoine Lacassagne Center, Côte d’Azur University, 31 Avenue de Valombrose, 06103 Nice, France; 2 Laboratory Transporter in Imaging and Radiotherapy in Oncology (TIRO), UMR E4320 TIRO-MATOs, Direction de la Recherche Fondamentale (DRF), Institut des Sciences du Vivant Fréderic Joliot, Commissariat à l’Energie Atomique et aux Energies Alternatives (CEA), Faculté de Médecine, Côte d’Azur University, 28 Avenue de Valombrose, CEDEX 2, 06107 Nice, France; 3 Department of Cytopathology and Anatomopathology, Antoine Lacassagne Center, 33 Av. de Valombrose, 06189 Nice, France; 4 Department of Radiology, Antoine Lacassagne Center, 33 Av. de Valombrose, 06189 Nice, France

**Keywords:** indeterminate thyroid nodules, fine-needle aspiration cytology, metabolomics, thyroid cancer, diagnostic biomarkers, machine learning, deep learning

## Abstract

Although it is the gold standard for assessing the malignancy of thyroid nodules (TNs) preoperatively, the cytological analysis of fine-needle aspiration cytology (FNAC) samples results in 20–30% of cases in indeterminate lesions (ITNs). As two-thirds of these lesions will appear benign after diagnostic surgery, improved preoperative diagnostic methods need to be developed. In this pilot study, we evaluate if the metabolomic profiles of liquid-based (CytoRich^®^) FNAC samples of benign and malignant nodules can allow the molecular diagnosis of TNs. We performed untargeted metabolomic analyses with CytoRich^®^ FNAC in a monocentric retrospective study. The cohort was composed of cytologically benign TNs, histologically benign or papillary thyroid carcinomas (PTCs) cytologically ITNs, and suspicious/malignant TNs histologically confirmed as PTCs. The diagnostic performance of the identified metabolomic signature was assessed using several supervised classification methods. Seventy-eight patients were enrolled in the study. We identified 7690 peaks, of which 2697 ions were included for further analysis. We selected a metabolomic signature composed of the top 15 metabolites. Among all the supervised classification methods, the supervised autoencoder deep neural network exhibited the best performance, with an accuracy of 0.957 (0.842–1), an AUC of 0.945 (0.833–1), and an F1 score of 0.947 (0.842–1). Here, we report a promising new ancillary molecular technique to differentiate PTCs from benign TNs (including among ITNs) based on the metabolomic signature of FNAC sample fluids. Further studies with larger cohorts are now needed to identify a larger number of biomarkers and obtain more robust signatures.

## 1. Introduction

Thyroid nodules (TNs) are found in two-thirds of individuals aged 20–60 and they are expected to become even more common in the coming years [1,2]. The medical community has witnessed a worldwide “epidemic” of thyroid cancers (TCs), which account for 5–10% of TNs, in recent decades [3,4]. However, according to the Bethesda System for Reporting Thyroid Cytopathology (TBSRTC) 25–30% of lesions are classified as Bethesda III (atypia/follicular lesions of undetermined significance) or IV (follicular/Hürtle cell neoplasms) after fine-needle aspiration cytology (FNAC), for which the distinction between benignity or malignancy is challenging [5,6]. In the presence of these cytologically indeterminate thyroid nodules (ITNs), patients are usually advised to undergo diagnostic surgery [6]. As these lesions are mostly benign, approximately 30% of thyroid surgeries for TNs could be avoided by a more accurate preoperative diagnosis, thus reducing unnecessary burdens on patients and health-care systems [3,4,6].

In the age of precision medicine, this situation started the race for the development and use of molecular tests to improve the diagnosis of ITNs. The 2015 recommendations of the American Thyroid Association included the use of molecular methods because of their high performance [7,8,9]. At that time, molecular tests aimed to identify TNs that could benefit from medical monitoring or surgery. These tests were based on the detection of genomic or transcriptomic alterations in TNs. More precisely, two different categories of molecular assays were designed based on opposite concepts. The first category includes “rule-in tests,” which aim to better identify the malignant lesions among the ITNs and to determine the extent of the surgery required. The second category includes “rule-out tests,” which aim to better identify benign lesions and thus prevent unnecessary surgeries. Today, the Thyroseq v3 and AFIRMA Genomic Sequencing Classifier are the most widely used tests, both considered to be at the same time “rule-in” and “rule-out” tests. The latest version of Thyroseq, v3 comprises targeted next-generation sequencing evaluation of point mutations, copy-number alterations, gene fusions, and abnormal gene expressions. Thyroseq v3 has been reported to reach 94% sensitivity and 82% specificity, with a negative predictive value (NPV) of 97% and a positive predictive value (PPV) of 66% [10]. The latest AFIRMA Genomic Sequencing Classifier is based on RNA sequencing and analysis of BRAFV600E mutations, RET/PTC1 or RET/PTC3, and specific alterations characteristic of parathyroid lesions, medullary thyroid cancer, and Hürtle cell lesions. The AFIRMA test has been reported to reach 94.3% specificity, an NPV of 100% and a PPV of 60%, with a benign call rate ranging from 63% to 76.2% [11,12]. Due to the respective efficiencies and underlying concepts of the molecular methods, the accuracy of posttest risk of malignancy for a given TN may vary according to the method used. Finally, from a technical point of view, these molecular methods frequently require at least one additional FNAC sample, the invasiveness of which is generally poorly tolerated by patients. Although these molecular methods are constantly being further developed and improved, they have serious limitations, which lie mainly in the high costs and time required for sample analysis [8]. Therefore, a new, fast, and cost-efficient approach that could also be automated in the near future is needed for more accurate routine TC diagnosis.

Metabolomics involves the large-scale study of thousands of small molecules present within cells, tissues, or biological fluids [13,14]. Cancer cells undergo specific metabolic reprogramming to meet their needs for growth and proliferation [15,16,17]. Unlike the current molecular tests, metabolomics focuses on the end products of these cellular regulatory processes and could therefore serve as a potent tool for thyroid nodule diagnosis. Nuclear magnetic resonance-based metabolomic analyses have already been demonstrated to be useful tools for the discrimination of TNs from adjacent healthy tissue and between benign and malignant lesions in surgical and FNAC specimens [18,19,20,21]. In addition, it has been shown that gas chromatography–mass spectrometry and LC-MS (liquid chromatography–mass spectrometry)-based metabolomic analyses are able to discriminate papillary thyroid carcinomas (PTCs) from healthy thyroid tissue [22,23].

In this pilot study, we aimed to evaluate the feasibility of metabolomic profiling based on untargeted LC-MS/MS and several supervised classification methods using liquid-cytology fluids from FNAC samples, specifically to discriminate malignant from benign TNs.

## 2. Materials and Methods

### 2.1. Patient Selection

We conducted a retrospective study based on data and samples of patients treated at the Institut Universitaire de la Face et du Cou (Nice University Hospital and Antoine Lacassagne Center) from 2019 to 2021. Clinical, ultrasonographical, cytological, and histological data were collected or extracted by the authors from computerized medical records.

All patients with Bethesda II–VI TNs that were subsequently removed (except for Bethesda II lesions) and then histologically diagnosed as a benign or malignant lesion were included in the study. Due to an insufficient number of cases to perform statistical analyses, patients with non-invasive follicular thyroid neoplasm with papillary-like nuclear features (NIFT-P) or non-PTC lesions were excluded from the cohort. Thus, the cohort consisted exclusively of patients with benign TNs (Bethesda II), ITNs (Bethesda III–IV) further identified as benign or PTCs on pathological examination, and suspicious/malignant TNs (Bethesda V–VI) that had been confirmed as PTCs on pathological examination.

### 2.2. Sample Collection, Cytological and Histological Diagnoses

Patients with TNs underwent FNAC according to the recommendations of the EU-TIRADS classification [24]. Cytological samples were obtained during ultrasound-guided FNAC, performed by a single experienced thyroid radiologist. For liquid-based cytology (LBC), thyroid cells were collected in CytoRich^TM^, centrifuged and spread onto a slide for staining and histological examination. The supernatants were collected and stored at 4 °C until metabolite extraction. Metabolite extraction and metabolomic analysis were generally performed within one month of FNAC when the patient was included in the study.

All samples were collected during routine practice and stored in the biological resource bank of the Antoine Lacassagne Center. The cytological and histopathological diagnoses were performed by thyroid cytologists and pathologists at the institution. For the cytological and histopathological classifications, the 2017 version of TBSRTC and the fourth edition of the World Health Organization classification of endocrine tumors were used [25].

### 2.3. Sample Preparation

FNAC samples: A quantity of 30 mL of CytoRich^TM^ was mixed with 90 mL of H_2_O (HPLC grade, Merck Millipore, Merck KGaA, Burlington, Massachusetts, USA). Metabolites were extracted using Solid Phase MicroExtraction (SPME) tips (Supelco, Bellefonte, PA, USA) according to the manufacturer’s protocol. Four SPME tips (two C18 SPME tips and two PDMS/DVB SPME tips from Supelco) were used for each sample. After incubation for 2 h at room temperature under constant agitation, SPME-extracted metabolites were eluted with 80 µL of a 20:80 acetonitrile-H_2_O mixture (HPLC grade, Merck Millipore, Burlington, MA, USA) and stored at −20 °C until use for LC-MS/MS analysis.

### 2.4. LC-MS/MS Analyses

Chromatographic analyses were performed with the DIONEX Ultimate 3000 HPLC system coupled with a chromatographic column (Phenomenex Synergi 4 u Hydro-RP 80A 250_3.0 mm) set at 40 °C and a flow rate of 0.9 mL/min. Gradients of mobile phases (mobile phase A: 0.1% formic acid in water and mobile phase B: 0.1% formic acid in acetonitrile) were performed for a total of 25 min. MS analysis was carried out on a Thermo Scientific Exactive Plus Benchtop Orbitrap mass spectrometer (Thermo Fischer Scientific Inc., Waltham, MA, USA). The heated electrospray ionization source (HESI II) was used in positive- and negative-ion modes. The instrument was operated in full scan mode from *m*/*z* 67 to *m*/*z* 1000. A quality-control sample was prepared from an equal mixture of ten benign samples and used to monitor the stability of the mass spectrometer performance.

### 2.5. Data Preprocessing and Statistical Analyses

The posttreatment of the obtained data was performed using MZmine2 version 2.39 (http://mzmine.github.io/, accessed on 28 April 2019) [26]. Mass detection was performed using the mass detector tool. Chromatograms were generated with the ADAP chromatogram builder. Peaks were separated using the Peak extender module. Retention times were normalized with the retention time calibration module. Peaks were then aligned using the join aligner algorithm with a tolerance of 10 ppm in *m*/*z* and 1 min in retention time. The Human Metabolome Database version 5.0 (HMDB, http://www.hmdb.ca, accessed on 7 January 2022) was used to predict ion identity with a mass tolerance of 15 ppm [27,28]. We only recorded ions predicted as [M + H]^+^ adducts in the positive mode and [M − H]^−^ adducts in the negative mode. The data were exported in csv format. We compiled the metabolites obtained from the positive and negative ionization modes into a single database.

Only metabolites with a mean peak intensity ≥10^4^ and a ratio of sample peak intensity to blank peak intensity of ≥50 were included in the final data set.

Statistical analyses of the untargeted metabolomic data were processed using statistical modules provided by MetaboAnalyst 5.0 (https://www.metaboanalyst.ca, accessed on 12 July 2022) [27]. Raw data were mean-centered, scaled, and logarithm-transformed. We first performed principal component analysis (PCA) to obtain an overview of the data set and then conducted a partial least squares-discriminant analysis (PLS-DA). The PLS-DA models were confirmed by cross-validation and the average variable importance in projection (VIP) scores were used to identify metabolites that differed in the sample classes. The PLS-DA is considered particularly suitable for the selection and interpretation of metabolite features when studying biological systems. Additional analyses were conducted using other supervised classification methods provided by MetaboAnalyst. We also used a new supervised autoencoder (SAE) that we recently developed and tested for metabolomics data [29]. This new procedure has the advantage of providing a confidence score for each prediction and offers a new efficient method for feature selection.

The raw MS data were also analyzed using Compound Discoverer 3.3 (Thermo Scientific France, Illkirch, France). MS and MS2 data of the top features were verified using Compound Discoverer. Only ions identified with both MZmine and Compound Discoverer were used for further analysis. The identities of the top features were manually verified by matching the obtained MS2 spectra with those of online data banks (MZCloud, HMDB, Metlin, or MassBank Europe (https://massbank.eu/MassBank/, accessed on 9 March 2020).

The performance of the SAE, support vector machine (SVM), PLS-DA, and random forest (RF) models was assessed in terms of accuracy, area under the receiver-operating characteristic (AUROC), precision, recall, and F1 score. A cross-validation method was used to evaluate the generalizability of each model.

### 2.6. Ethical Aspects

Our research was created in accordance with the Declaration of Helsinki. Our protocol was approved by the Local Protection Committee of the Antoine Lacassagne Center of Nice. All samples belonged to the biobank of the Pathology Department of the Antoine Lacassagne Center and were subject to registration by the French Health Ministry, which regulates the biobank. This study meets the requirements of the reference methodology MR-004 of the Commission Nationale de l’Informatique et des Libertés (deliberation 2018-155, 3 May 2018) and was declared under number MR 2230368v0. As requested by MR04 and RGPD regulation, all eligible patients were informed of the use of their data for this retrospective observational study and were allowed to decline to participate in hindsight.

## 3. Results

### 3.1. Database Construction of the FNAC Metabolomic Analyses

**Figure 1:**
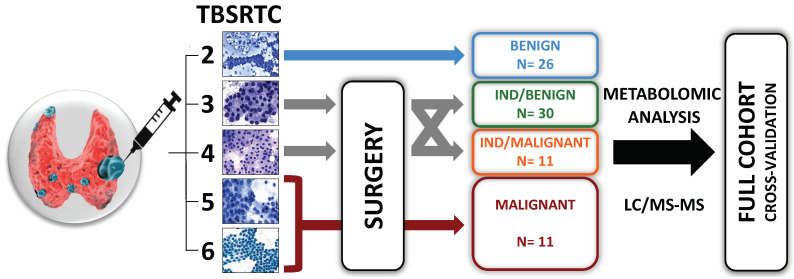
Graphical representation of the flowchart of the study. Patients with thyroid nodules (TNs) underwent ultrasound-guided fine-needle aspiration cytology (FNAC) according to the recommendations of the EU-TIRADS classification [24]. Patients were managed in accordance with the Bethesda system for reporting thyroid cytopathology (TBSRTC) and the ATA recommendations [13,14]. For Bethesda 2 lesions (i.e., benign lesions), no surgery was performed, except for compressive nodules. For other cytology results, only patients who underwent surgery with a histological diagnosis of benign or malignant lesion were included. Patients with TNs with two consecutive Bethesda 3 cytology results or with Bethesda 4 lesions (i.e., indeterminate TNs) were referred to diagnostic surgery. Patients with Bethesda 5 or 6 lesions (i.e., suspicious or malignant lesions) were referred to surgery. Metabolomic analysis with liquid chromatography-coupled mass spectrometry (LC-MS/MS) was performed on the supernatants of liquid-based FNAC collected in CytoRich^TM^.

Originally, 90 patients who met the inclusion criteria were enrolled in the study. It was later determined that four patients had a NIFT-P lesion and that three TC patients had non-PTC lesions (1 follicular TC, 1 oncocytic TC and 1 medullary TC). We decided to exclude the samples from these patients because the small number of each histological type made robust statistical analyses impossible. The samples from five patients were degraded and were therefore also excluded. The total cohort finally comprised 78 patients with TNs: 26 benign TNs (Bethesda II), 30 benign and 11 PTC cytologically indeterminate (Bethesda III–IV), and 11 PTC cytologically suspicious/malignant (Bethesda V–VI). The graphical representation of the experimental flowchart is shown in Figure 1. The demographic, biological, ultrasonographical, cytological, and histological characteristics of the patients and samples are summarized in Table 1, and fully described in Supplementary Tables S1–S4. Briefly, the cohort included 74.4% women, aged 52.6 years (±15.8), mainly euthyroidic (83.3%), with a TN of diameter 23.1 mm (±10.4). Patients with benign TNs presented an adenoma or a non-neoplastic nodule in 50% of the cases. Among the patients with a PTC, 54.5% of the cases were of a classical variant.

### 3.2. FNAC Metabolomic Analyses Allowed Preoperative Diagnosis of Thyroid Nodules

**Figure 2:**
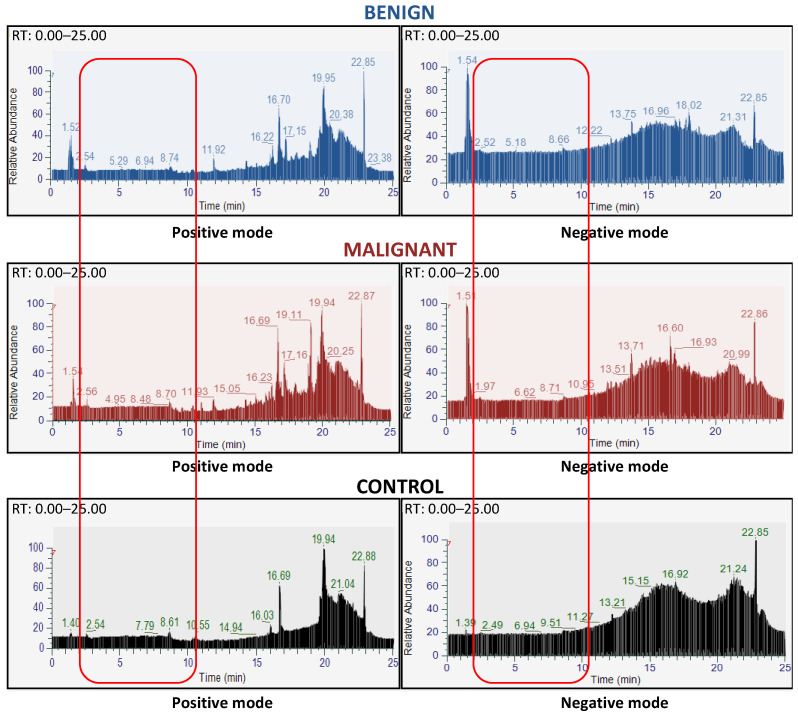
Metabolomic analyses were feasible on liquid-based cytology of thyroid nodules. Representation of liquid chromatography spectra of a benign sample (blue upper panels), a malignant sample (dark-red middle panels), and a control (dark lower panels) in the positive (left panels) and negative mode (right panels), respectively. The light-red rectangles highlight the absence of hydrophilic metabolites (between 2 and 10 min of retention time) found in the positive and negative modes, in both benign and malignant nodules. Within a large background, there is no clear difference in the chromatography spectra between benign and malignant nodules. The chosen spectra are representative of the sample set for each class. The x-axis represents the retention time, and the y-axis represents the relative intensity of the peaks.

In the 78 SPME-purified FNAC samples, we identified 5081 and 2609 peaks in the positive and negative modes, respectively, using MZmine. Among these, 2282 and 815 peaks could be putatively identified by matching the *m*/*z* with the [M + H]^+^ or [M − H]^−^ of metabolites from the HMDB, in the positive- and negative-ion modes. As expected from the use of C18 and PDMS/DVB SPME, and as observed in the technical development experiments, sample preparation led to a loss of some non-polar metabolites with low retention times in both benign and malignant samples. Figure 2 shows a representative total ion chromatogram (TIC) of benign samples, malignant samples and blanks (acetonitrile solution only) for electrospray ionization in negative and positive modes. It can be observed that the intensities of specific signals were low and only a few peaks of the samples could be clearly distinguished in the TIC from those of the blanks. However, these low signal intensities were to be expected due to the small volume of the biological samples, i.e., small tissue section (FNAC), and the loss of ions due to the CytoRich™/SPME purification method. Several specific data evaluation steps were performed to select accurate ions. In a first step, in order to reduce the background noise, a minimum value was set for the mean of the signal intensities of all samples. In a second step, only ions with a 50-fold higher peak intensity in the samples compared to the blanks were retained. After these two steps, the data set still comprised 2697 ions, which were further analyzed.

A series of statistical analyses was then performed to identify the most relevant metabolites based on the VIP scores of the PLS-DA model. Of the top metabolites, those which did not show clear MS1 and MS2 spectra with Compound Discoverer were eliminated. Subsequently, a new analysis was performed using the same statistical procedure described above (see Section 2), but unfortunately it was not possible to discriminate benign from malignant samples with the unsupervised PCA method (Supplementary Figure S1A). The performance of the PLS-DA model showed satisfactory accuracy, but resulted in poor “goodness of prediction” (Q2 score) and high “goodness of fit” (R2 score), suggesting an overfitting (Supplementary Figure S1B–D). However, the same analysis performed with SAE allowed discrimination between benign and malignant tissue in the latent space of nearly all samples (Supplementary Figure S1E). The classification of the sample was uncertain in only a few cases, as indicated by the SAE kernel density results (Supplementary Figure S1F). Our results suggest that SPME-tip purification of CytoRich ™-based thyroid FNAC samples enables metabolomic analysis for preoperative diagnosis of thyroid nodules, even though observed signal intensities were low and some metabolites were probably subject to partial chemical alterations.

### 3.3. Metabolomic Profiling with 15 Features from FNAC Samples Allowed Preoperative Diagnosis of Thyroid Nodules

**Figure 3:**
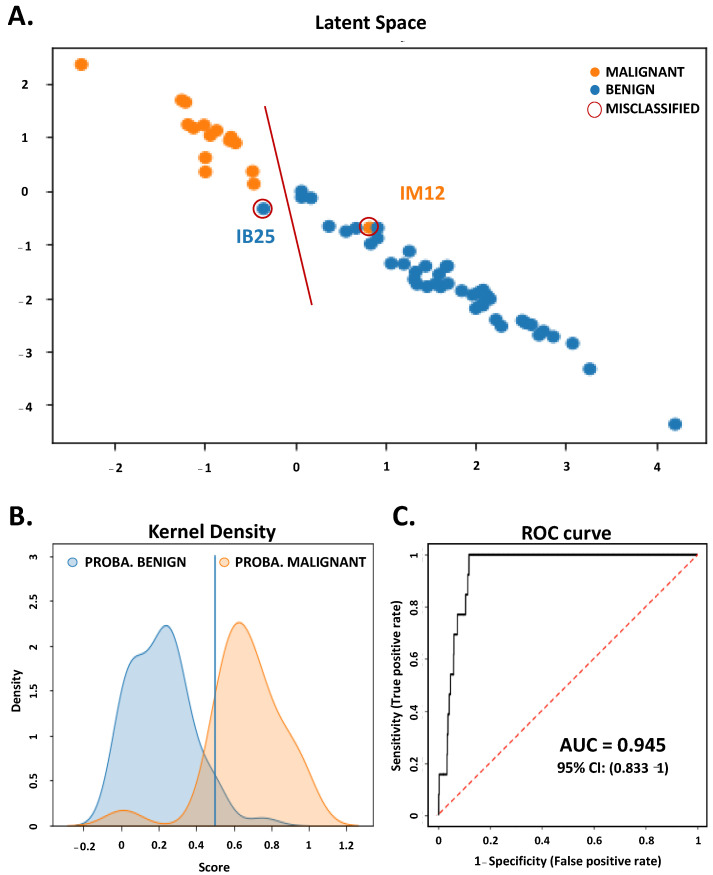
A supervised autoencoder can accurately distinguish benign from malignant thyroid nodules using a liquid-based FNAC signature of 15 metabolites. A deep neural network (the SAE) was used to perform statistical analysis on the dataset [9]. From the initial database, it was possible to identify 15 relevant and statistically significant metabolites using the SAE. This 15-metabolite signature was used thereafter. (**A**) Distinct clustering of benign (blue dot) and malignant samples (orange dot) in the two dimensions of the latent space. The samples that were misclassified are annotated and encircled in red. (**B**) shows the kernel density estimation of the prediction probability in benign (blue) and malignant classes (orange). The kernel density is presented as a function of the prediction probability score. The smooth curve representation of histograms in this figure allows a better visualization. (**C**) Area under the curve (AUC) of the receiver-operating characteristic (ROC) of the metabolomic signature identified by the SAE and its 95% confidence interval. The sensitivity (true-positive rate) was expressed as a function of 1-specificity (false-positive rate).

We then wanted to reduce the number of metabolites to get the most parsimonious signature, using the SAE model with a step-by-step top-down procedure (Supplementary Figure S2). We obtained a short list of the top 15 ions of the SAE model. After logarithmic normalization, these 15 ions made it possible to accurately discriminate nearly all benign from malignant TNs. The performance of the SAE model is illustrated by the latent space for benign versus malignant samples (Figure 3A), and by a separation of the two curves of the kernel density estimation of the prediction probability (Figure 3B). Only two samples, one in each category, were misclassified by the SAE model. The misclassified samples (IB25 and IM12) are marked in Figure 3A. Using these top 15 ions, the SAE model yielded an accuracy of 0.957 (0.842–1), an AUC of 0.945 (0.833–1), a precision of 0.957 (0.864–1), a recall of 0.945 (0.833–1) and an F1 score of 0.947 (0.842–1) (Figure 3C, Table 2 and Table 3). Remarkably, the performance of the SAE model was maintained throughout the 12-fold cross-validation analysis, with only minor variations between the individual cross-validations.

**Figure 4:**
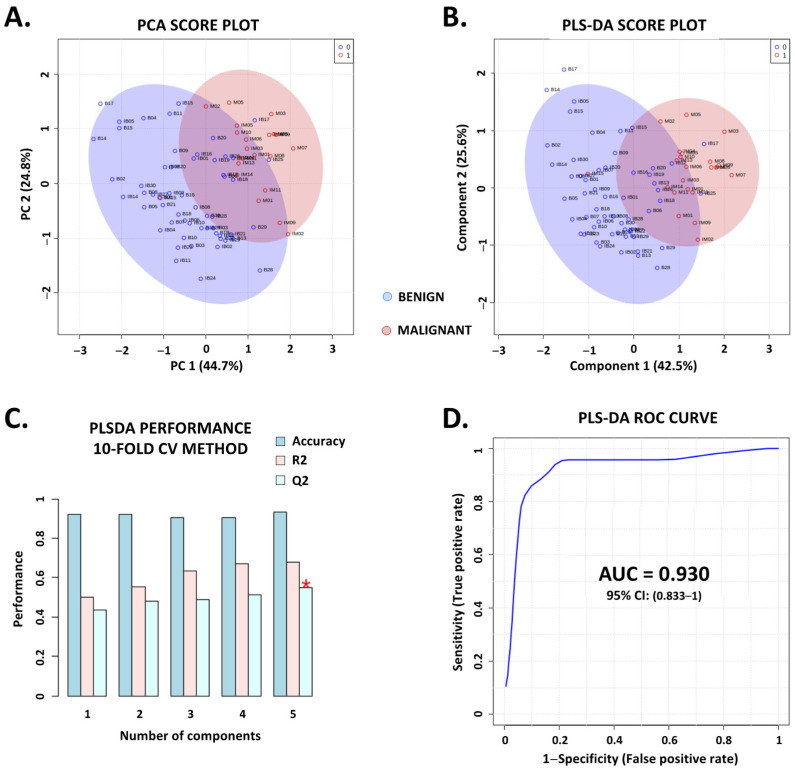
Conventional multidimensional machine learning algorithms support the validity and efficiency of the metabolomic signature. Based on the 15-metabolite signature identified through the supervised autoencoder, we conducted more classical multidimensional machine learning analysis to increase the validity. A principal component analysis (PCA) and a partial least squares-discriminant analysis (PLS-DA) were performed using the univariate and multivariate analysis modules provided by MetaboAnalyst 5.0. (**A**,**B**) PCA and PLS-DA (respectively) score plots, built with the two first components that illustrate a tendency to distinguish between benign (blue dots) and malignant thyroid nodules (red dots). (**C**,**D**) show the performance of the PLS-DA model in terms of accuracy (0.934 (0.895–1)), goodness of fit (0.68, R2 score), and goodness of prediction (0.591, Q2 score) validated with a 10-fold cross-validation (CV) method (**C**) and the area under the curve (AUC) of the receiver-operating characteristic (ROC) and its 95% confidence interval (**D**). For figure C, the red star indicates the model with the best R2 score (5 components PLS−DA model). For the AUROC curve, the sensitivity (true-positive rate) was expressed as a function of 1-specificity (false-positive rate).

The identity (main identity, HMDB identification, and molecular formula), the mean peak benign/malignant ratio, and the weight of each metabolite within the model constructed by the SAE (in descending order) are shown in Table 4. We also studied the performance of other classification methods using the same top 15 ions. While the unsupervised PCA method could only reveal a tendency to distinguish between benign and malignant samples (Figure 4A, Supplementary Figure S3), the supervised PLS-DA model reached an accuracy of 0.934 (0.895–1), an AUC of 0.93 (0.833–1), a F1 score of 0.903 (0.802–1), a goodness of fit (R2 score) of 0.68 and a goodness of prediction (Q2 score) of 0.591 (Figure 4B–D, Table 5). The permutation tests demonstrated that the model was robust and reproducible, with *p*-values < 0.05 after 2000 permutations (Supplementary Figure S4).

Finally, the performances of both the supervised SVM and the RF model were also consistent with those described above, showing accuracies of 0.908 (0.842–0.947) and 0.947 (0.895–1), AUCs of 0.875 (0.771–0.958) and 0.953 (0.887–1), and F1 scores of 0.873 (0.737–0.945) and 0.936 (0.887–1), respectively (Table 5). These performances remained constant throughout a fourfold cross-validation test. The importance of each metabolite was (slightly) different depending on the classification method used and could be the reason for the small differences in the performances achieved by the different models (Supplementary Table S5).

## 4. Discussion

Here, we report LC-MS/MS metabolomic analyses aiming to improve the diagnosis of TCs among ITNs. We show that this approach enabled the identification of new metabolomic signatures for more accurate TN diagnosis. We demonstrated that in the case of ITNs, a metabolomic signature using liquid-based cytology could distinguish benign from malignant lesions. Thus, the LC-MS/MS-based metabolomic approach offers a new ancillary technique for the evaluation of ITNs, providing a rapid and cost-efficient alternative to the currently available molecular tests [9]. In the era of the search for a therapeutic de-escalation of TC, the metabolomic approach is an alternative option to reduce the number of unnecessary surgeries and the global medico-economic impact of ITN management.

Thyroidectomy is the main component in the management of thyroid cancer. Although the procedure is generally considered safe, it also carries potential surgical complications, especially in the case of total thyroidectomy. The more extensive surgical procedures for thyroid cancer, which often require removal of lymph nodes and resection of potentially aggressive tumor material, contribute to an increased risk of complications. Specific postoperative complications of thyroid cancer surgery include transient or permanent injury of the laryngeal nerve and the occurrence of hypoparathyroidism. The usually temporary injury of the recurrent laryngeal nerve leads to hoarseness or swallowing difficulties and occurs in 4.5% of cases after total thyroidectomy and in 3.3% of cases after hemithyroidectomy [30]. Although hypoparathyroidism is usually also transient, it can be permanent, occurring in 21.3% of cases after total thyroidectomy and in 1.8% of cases after total thyroidectomy [30]. Other non-specific complications include hematomas, wound infections, impaired wound healing, excessive bleeding, and damage to the surrounding nerves. These complications remain relatively rare, but when they do occur, they have a significant impact on the quality of life of the affected patients. It is therefore necessary to develop an innovative approach to classify indeterminate thyroid nodules, and thus to reduce unnecessary burdens on patients and health-care systems.

The first main challenge of this study was to establish a protocol for the extraction of metabolites from the CytoRich^TM^ solution of FNAC samples. In our experience, liquid-phase extraction methods do not result in good separation of the metabolites from the fixatives of the CytoRich^TM^ solution, so sample preparation for LC-MS/MS analysis was not possible. We therefore tested several approaches based on solid-phase extraction methods. The best sample preparations for LC-MS/MS analyses were achieved with SPME tips. Despite our promising results, more experiments are still needed to further improve the reproducibility of metabolite extraction using SPME tips.

In this study, given the very small amount of biological material available (due to FNAC), a single SPME extraction was performed per sample. This extract was used entirely for two LC-MS/MS analyses, i.e., one in positive and one in negative mode. As expected, the MS signals were low, but a large number of ions could be detected. For best reproducibility and sensitivity, the LC-MS/MS analyses of all the samples shown in this study were performed consecutively in a single run. The order in which the samples were subjected to LC-MS/MS analyses was based on their entry number in the biobank and was thus determined randomly. Our standard procedure included regular controls of all MS parameters and equipment throughout the whole acquisition (pump pressure, quality of the spectra, regular prefilter replacement, etc.). No significant variations in the analyses were found with pooled samples from several subjects that were carried out as quality controls. Blank samples were included to subtract background noise from signals (see Results). In addition, we verified that the peaks of the individual ions determined with MZmine could also be identified with another posttreatment software of MS/MS data, Compound Discoverer. Furthermore, this second software predicted if the ion was [M + H]^+^ for the positive mode or [M − H]^−^ for the negative mode, another adduct, or an isotope (see Supplementary data). The reproducibility of the obtained data was an important issue in this study. These first analyses with small sample sets already indicated the importance of the experimental procedures and sample collection for reproducible identification of the top ions. However, the results obtained are promising, but further studies with a larger number of samples should now be performed to strengthen the robustness of the metabolic signature. Special attention should be paid to the standardization of sample storage after FNAC. In addition, a special procedure should be developed to remove CytoRich^TM^ fixatives prior to SPME extraction and LC-MS/MS analysis. Gas chromatography (GC-MS/MS), another MS-based method that may be less sensitive to contaminants from the CytoRich^TM^ solution, and lipidomic analysis could be performed.

With the ever-increasing prevalence of ITNs, the molecular tests already available are being used more and more frequently [31]. Despite the good diagnostic performance of these tests, their high cost remains a challenge for health-care systems worldwide [8]. New methods are constantly emerging to provide better patient management [20,32,33]. Our aim was to identify a metabolomic signature that would allow accurate, rapid and cost-effective discrimination between malignant and benign ITNs using LBC fluid, thus avoiding the burden of additional biopsies on the patient. As described above, we first processed the database by filtering and denoising the data to retain only the most intense, frequent, and statistically significant metabolites. In addition, we used robust supervised machine learning and deep learning algorithms to perform statistical analyses on multiple samples, which allowed us to accurately diagnose benign and malignant ITNs (Figure 3 and Figure 4, Table 2, Table 3 and Table 4). The deep learning (SAE) algorithm provided the best performance for a model classifying benign and malignant nodules in terms of accuracy and AUC. In the context of unbalanced data, the obtained F1 score was also a metric to be emphasized. By combining two competing metrics (precision and recall scores), the SAE approach assesses the predictive ability of a model by determining its class-specific performance rather than its overall performance, as is the case when calculating accuracy. Regarding the F1 score, the SAE was still the best classification method compared to all the other methods tested. The consistent performance of the different algorithms tested strongly supported the relevance of the metabolomic signature identified. As mentioned above, the metabolomic analysis was performed with the CytoRich^TM^ solution remaining after cytological examination of the FNAC sample. Therefore, the approach can be used for all cytological ITNs without requiring an additional biopsy.

Previous studies have shown the usefulness of identifying metabolomic signatures to distinguish TCs from benign TNs or healthy tissue. However, to our knowledge, this is the first study to determine an LC/MS-based metabolomic signature using LBC samples to diagnose ITNs. Several groups describe alterations of metabolic pathways found in tissue samples from TCs that are also detectable in serum, plasma or whole-blood samples from these patients [34,35,36,37]. Feng et al. and Zhang et al. report metabolic differences in the urine and feces of patients with papillary TC and healthy volunteers [36,38]. Rezig and collaborators describe a high-resolution magic angle spinning magnetic resonance spectroscopy (HRMAS-MRS) approach on ex-vivo FNAC samples collected from postsurgical thyroid nodules. The authors show that although NMR spectral features allow the differentiation between follicular adenomas and papillary thyroid carcinomas, diagnostic accuracy is only 84.8% when samples from ITNs are analyzed [39].

Although our approach enabled the determination of a specific signature for ITNs with high accuracy, many metabolites of the FNAC samples could not be identified. Storage at 4 °C in the CytoRich^TM^ solution may have led to the chemical transformation of many metabolites, which then made the identification of ions produced during LC-MS/MS impossible. While this did not compromise the discriminatory performance of the metabolomic approach, it was more difficult to gain insights into the underlying biological processes. Further strategies need to be developed to reduce the impact of the CytoRich^TM^ solution to improve the identification of relevant metabolites and consequently the quality of the analyses. In addition, due to the small number of patients with follicular TC, oncocytic TC or NIFT-P CT in our initial cohort, we had to exclude samples from these patients from our study to ensure robust statistical analyses. In a future prospective study, we will try to identify a metabolomic signature that will allow the differentiation of these lesions from benign nodules and also from PTCs.

## 5. Conclusions

Today, the main goal in the management of TC and its global medico-economic impact is the reduction in cost-intensive molecular tests and unnecessary thyroidectomies. Therefore, the accurate diagnosis of ITNs is currently one of the biggest challenges in the field. In this pilot study, we used LC-MS/MS for metabolomic analyses of FNAC samples to identify novel diagnostic biomarkers and a metabolomic signature for ITN diagnosis. We were able to identify a metabolomic signature that allowed us to distinguish between malignant and benign nodules among the ITNs, although we analyzed only the small amount of FNAC fluid that remained after cytology. Further analyses with a larger number of samples are now required to obtain an even more robust signature and to implement the approach in routine clinical practice.

## Figures and Tables

**Table 1:** Demographic characteristics of the cohort of patients.

Characteristics			All % (nb)	Benign % (nb)	Malignant % (nb)
			78	56	22
Gender		F	74.4 (58)	76.8 (43)	68.2 (15)
Age (mean ±SD)			52.6 (±15.8)	56.1 (±14.6)	43.7 (±15.4)
Hashimoto			11.4 (8)	6.3 (3)	22.7 (5)
Thyroid function		Euthyroidism	83.3 (65)	82.1 (46)	86.4 (19)
		Hypothyroidism	7.7 (6)	7.1 (4)	9.1 (2)
		Hyperthyroidism	7.7 (6)	8.9 (5)	4.5 (1)
EU-TIRADS		3	23.1 (18)	32.1 (18)	0
		4	46.2 (36)	48.2 (27)	36.4 (8)
		5	29.5 (23)	17.9 (10)	59.1 (13)
US size (mm, mean ±SD)			23.1 (±10.4)	25.4 (±10.2)	17.4 (±8.9)
Cytologic classification (Bethesda)		II	33.3 (26)	46.4 (26)	0
		III	20.5 (16)	17.9 (10)	27.3 (6)
		IV	32.1 (25)	35.7 (20)	22.7 (5)
		V	6.4 (5)	0	22.7 (5)
		VI	7.7 (6)	0	27.3 (6)
Histology	Benign	NNN	30.4 (17)	50 (17)	0
		Adenoma	30.4 (17)	50 (17)	0
	Malignant (PTC)	Classical variant	15.4 (12)	0	54.5 (12)
		Follicular variant	6.4 (5)	0	22.7 (5)
		Sclerosant variant	2.6 (2)	0	9.1 (2)
		Oncocytic variant	1.3 (1)	0	4.5 (1)
		Tall cell variant	1.3 (1)	0	4.5 (1)
		Solid variant	1.3 (1)	0	4.5 (1)

Data are % (nb), or mean (±standard deviation). Abbreviations: F, female; NNN, non-neoplastic nodule; PTC, papillary thyroid carcinoma. Bethesda classification: II (benign lesion); III (atypia of undetermined significance, AUS); IV (FN, follicular neoplasm; HCN, Hürtle cell neoplasm; sFN, suspicious for follicular neoplasm); V (suspicious for malignancy); VI (malignant).

**Table 2:** Predictive capability of the Autoencoder.

Folds	Global	Benign	Malignant
Fold 1	1	1	1
Fold 2	0.95	1	0.667
Fold 3	0.95	1	0.875
Fold 4	0.842	0.727	1
Fold 5	1	1	1
Fold 6	0.95	0.933	1
Fold 7	0.9	0.933	0.8
Fold 8	1	1	1
Fold 9	1	1	1
Fold 10	1	1	1
Fold 11	0.95	1	0.833
Fold 12	0.947	0.917	1
**Mean**	**0.957**	**0.959**	**0.931**
SD	0.047	0.077	0.107

Abbreviations: SD, standard deviation.

**Table 3:** Performance of the Autoencoder.

Folds	AUC	Precision	Recall	F1 Score
Fold 1	1	1	1	1
Fold 2	0.833	0.972	0.833	0.886
Fold 3	0.938	0.962	0.938	0.947
Fold 4	0.864	0.864	0.864	0.842
Fold 5	1	1	1	1
Fold 6	0.967	0.917	0.967	0.937
Fold 7	0.867	0.867	0.867	0.867
Fold 8	1	1	1	1
Fold 9	1	1	1	1
Fold 10	1	1	1	1
Fold 11	0.917	0.967	0.917	0.937
Fold 12	0.958	0.938	0.958	0.945
**Mean**	**0.945**	**0.957**	**0.945**	**0.947**
SD	0.059	0.049	0.059	0.054

Abbreviations: AUC, area under the receiving-operator characteristic curve; SD, standard deviation.

**Table 4:** Importance and identity of the 15 top metabolites.

Ion Mode	Row m/z	RT (min)	Main Identity	Row Identity (HMDB)	Molecular Formula	SAE Score	B/M Ratio
Neg	434.872	1.41	-	-	C11H3O13P3	0.9726	7.002
Pos	307.083	2.56	(-)-Epigallocatechin	0038361	C15H14O7	0.7123	4.831
Pos	229.041	10.88	Indolylmethylthiohydroximate	-	C10H10N2OS	0.3305	3.067
Pos	217.08	2.56	-	-	-	0.3208	3.125
Pos	229.954	2.01	-	-	-	0.2623	0.554
Pos	247.965	2.02	-	-	-	0.1823	0.565
Pos	257.949	2	-	-	-	0.1790	0.573
Pos	311.9	1.78	-	-	-	0.1590	0.602
Pos	325.942	2	-	-	-	0.1495	0.590
Pos	210.933	1.7	-	-	-	0.1353	0.686
Pos	253.91	1.51	-	-	-	0.1336	0.612
Pos	162.907	1.49	Phosphoroselenoic acid	0003840	H3O3PSe	0.1329	0.682
Pos	225.914	1.51	-	-	-	0.1154	0.634
Neg	108.901	1.65	-	-	-	0.0247	1.731
Pos	250.939	1.41	-	-	-	−0.0289	5.156

Abbreviations: B/M, Benign over malignant ratio; HMDB, Human Metabolome Database; Pos, positive mode; Neg, negative mode; RT, Retention time; SAE, Supervised autoencoder.

**Table 5:** Performance of the machine learning methods.

Methods Performance		Fold 1	Fold 2	Fold 3	Fold 4	Mean	SD
Support Vector Machines	AUC	0.833	0.771	0.938	0.958	**0.875**	0.088
	Accuracy	0.895	0.842	0.947	0.947	**0.908**	0.050
	Precision	0.833	0.771	0.938	0.958	**0.875**	0.088
	Recall	0.933	0.717	0.958	0.938	**0.886**	0.114
	F1 score	0.864	0.737	0.945	0.945	**0.873**	0.098
	Time elapsed	0.025	0.029	0.028	0.029	**0.028**	-
Partial Least Squares- Discriminant Analysis	AUC	0.833	0.941	1	0.944	**0.930**	0.070
	Accuracy	0.895	0.895	1	0.947	**0.934**	0.050
	Precision	0.833	0.941	1	0.944	**0.930**	0.070
	Recall	0.933	0.750	1	0.955	**0.909**	0.110
	F1 score	0.864	0.802	1	0.947	**0.903**	0.088
	Time elapsed	0.004	0.001	0.001	0.001	**0.002**	-
	sil_plsda	0.396	0.435	0.407	0.381	**0.405**	0.023
	AUC	1	0.969	0.887	0.958	**0.953**	0.048
	Accuracy	1	0.947	0.895	0.947	**0.947**	0.043
Random	Precision	1	0.969	0.887	0.958	**0.953**	0.048
Forest	Recall	1	0.875	0.887	0.938	**0.925**	0.057
	F1 score	1	0.912	0.887	0.945	**0.936**	0.049
	TimeElapsed	0.362	0.343	0.344	0.342	**0.348**	-

Abbreviations: AUC, area under the receiving-operator characteristic curve; SD, standard deviation.

## Data Availability

The anonymized data dictionary will be made available on request by email to the main authors, after approval of a proposal and with a signed data access agreement.

## Supplementary Materials

The following supporting information (4 supplementary figures, 5 supplementary tables) can be downloaded at https://www.mdpi.com/article/10.3390/metabo13070782/s1. Figure S1: Machine learning and deep learning methods enabled identification of the most relevant and significant metabolites; Figure S2: A method for progressive identification of the metabolomic signature of interest with the supervised autoencoder; Figure S3: Details of the unsupervised principal component analysis model used on the 15-metabolite signature with MetaboAnalyst 5.0; Figure S4: Details of the supervised partial least squares-discriminant analysis model used on the 15-metabolite signature with MetaboAnalyst 5.0 Table S1: Demographic characteristics of patients with Bethesda II nodules; Table S2: Demographic characteristics of patients with benign Bethesda III–IV nodules; Table S3: Demographic characteristics of patients with malignant Bethesda III–IV nodules; Table S4: Demographic characteristics of patients with malignant Bethesda V–VI nodules; Table S5: Importance of metabolites in function of different algorithms.
