# Supplementary material for: Pilot Study on the Use of Untargeted Metabolomic Fingerprinting of Liquid-Cytology Fluids as a Diagnostic Tool of Malignancy for Thyroid Nodules
Source: Metabolites. 2023 Jun 23;13(7):782. doi: 10.3390/metabo13070782 (PMC10384948; doi:10.3390/metabo13070782)
Supplement: Supplementary file 1 [file metabolites-13-00782-s001.zip › metabolites-2423628_supplementary.pdf]

SUPPLEMENTARY DATA

SUPPLEMENTARY FIGURES

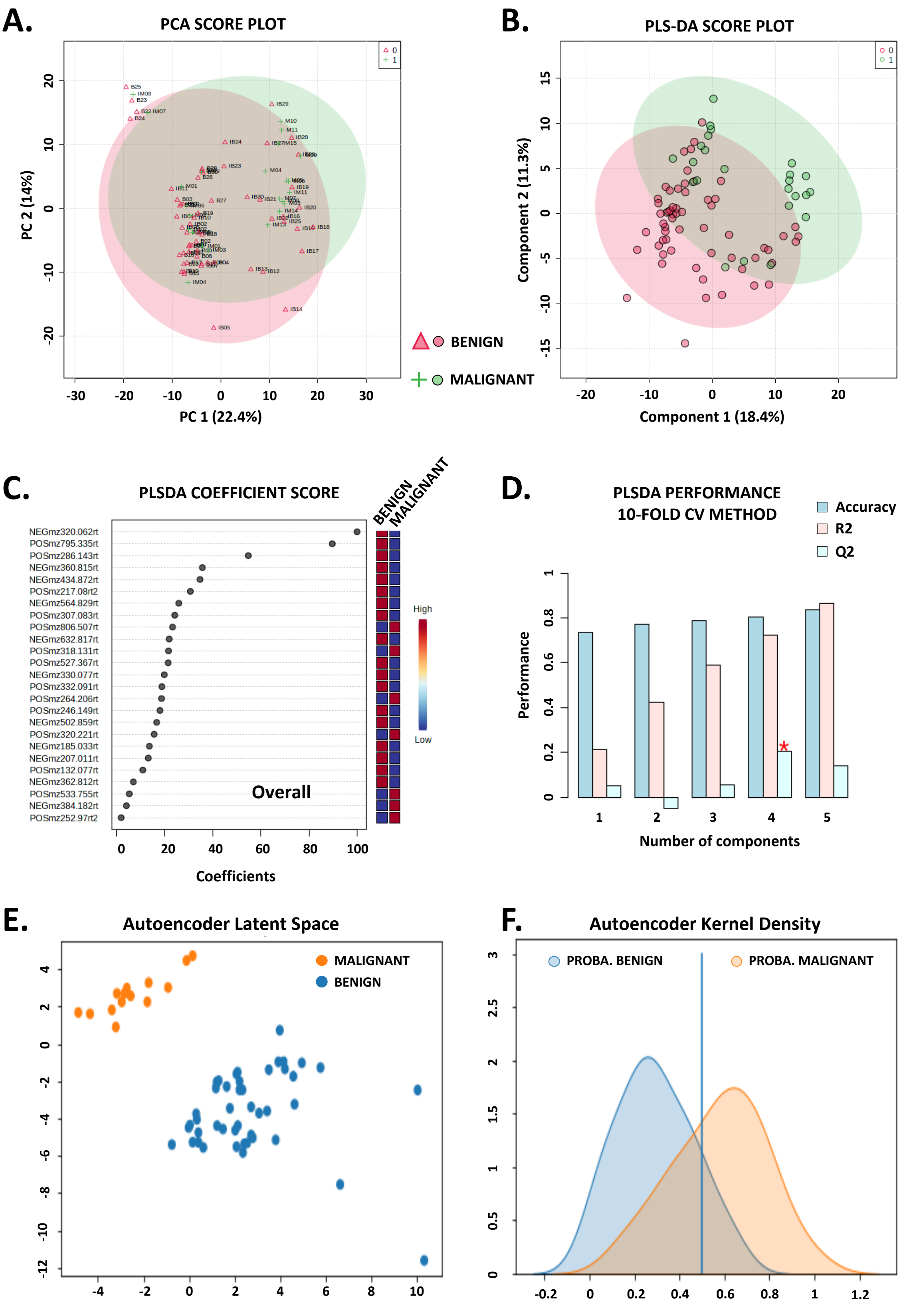

Supplementary Figure S1: Machine learning and deep learning methods enabled identification of the most relevant and significant metabolites.

We wanted to identify the most parsimonious metabolomic signature through a series of robust statistical analysis conducted on the database. We first performed unsupervised principal component analysis (PCA score plot - **Figure S1A**), and then a partial least squares-discriminant analysis (PLS-DA score plot - **Figure S1B**). While the PCA did not show any distinction between the two classes of samples, the PLS-DA showed a slight tendency to distinguish between benign (***red triangles/dot***) and malignant samples (***green triangles/dot***). The overall PLS-DA coefficient score of the 25 most important metabolites is shown in **Figure S1C**. In the right column, the relative concentration of the metabolite is represented in blue when reduced or in red when augmented, in benign (***left column***) or malignant (***right column***) thyroid nodules. **Figure S1D** shows the PLS-DA performance using a 10-fold cross-validation method in terms of accuracy (***blue columns***), "goodness-of-prediction" (Q2 score – ***light blue columns***), and "goodness-of-fit" (R2 score – ***pink columns***). We then used a deep learning supervised autoencoder to help in the selection of the metabolites. **Figure S1E** shows a distinct clustering of benign (***blue dot***) and malignant samples (***orange dot***) in the two dimensions of the latent space, although they are scattered in a large point cloud. **Figure S1F** shows a distinction on average between the prediction probabilities of benign (***blue***) versus malignant samples (***orange***) with a Kernel density estimation graphic. The Kernel density is presented as a function of the score of prediction probability. The smooth curve representation of histograms in this figure allows a better visualization.

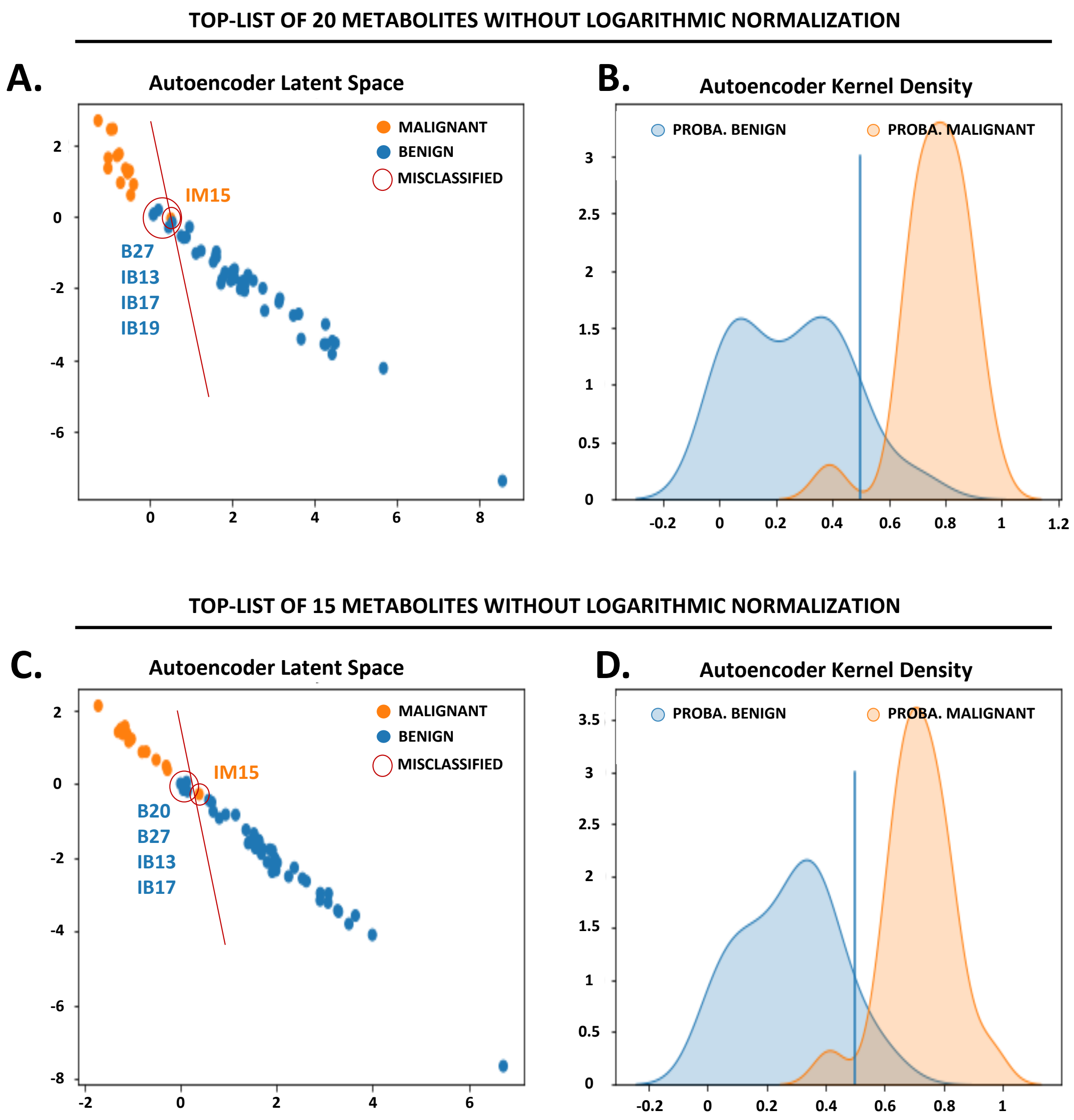

Supplementary Figure S2: A method for progressive identification of the metabolomic signature of interest with the supervised autoencoder.

We then wanted to reduce the number of metabolites to get the most parsimonious signature, using the supervised autoencoder model with a step-by-step top-down procedure. **Figures S2A** and **S2B** show the results obtained based on a signature of the top 20 ions, while **Figures S2C** and **S2D** show the results obtained based on the top-15 ions signature, both without logarithmic normalization. **Figures S2A** and **S2C** show the results of the latent space, which remains tight (artificially symbolized by a ***red bar***) but with a progressive separation of the two clusters of

benign (***blue dot***) and malignant samples (***orange dot***) with the reduction in the number of metabolites. Misclassified samples are ***encircled in red***.

Figures **S2B** and **S2D** show a distinction on average between the prediction probabilities of benign (***blue***) versus malignant samples (***orange***) with a Kernel density estimation graphic. The Kernel density is presented as a function of the prediction probability score. The smooth curve representation of histograms in this figure allows a better visualization. We can see that the flattened dome of the prediction probability of benign lesions tends to get thinner to form a peak with the reduction in the number of metabolites.

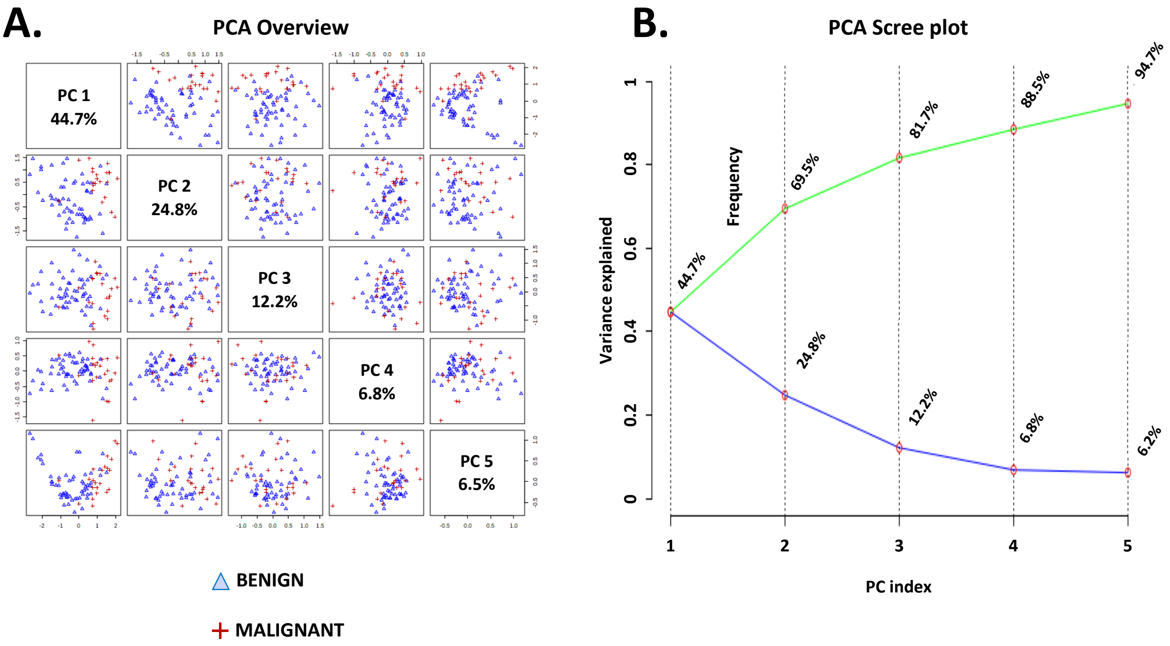

Supplementary Figure S3: Details of the unsupervised principal component analysis model used on the 15-metabolite signature with MetaboAnalyst 5.0

Figure S3A shows an overview of the principal component analysis (PCA) score plots according to the different components. Regardless of the components that may be involved, the PCA is unable to distinguish benign (***blue triangles***) from malignant samples (***red crosses***). The PCA scree plot (**Figure S3B**) shows the evolution of the variance explained according to the number of components involved.

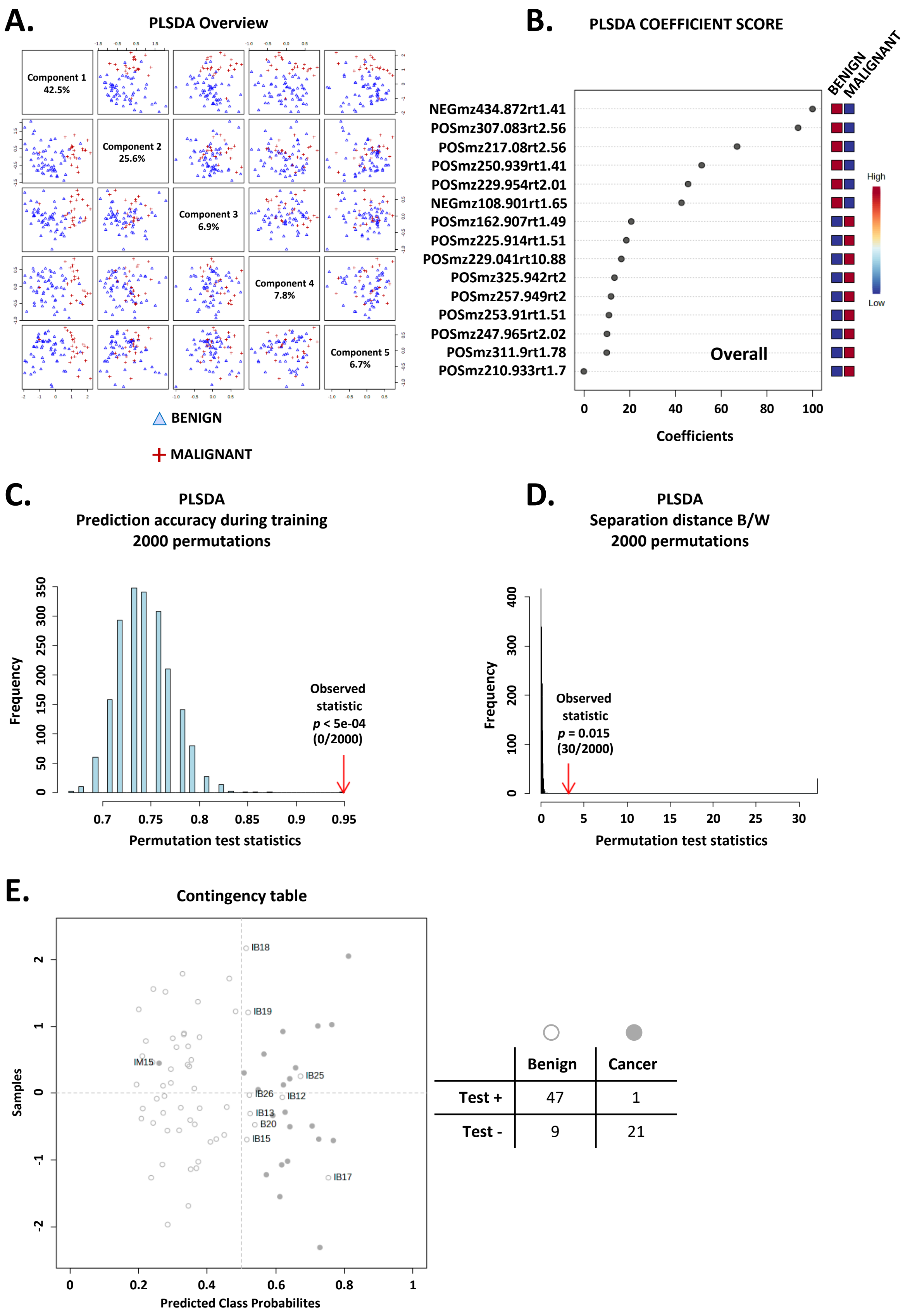

	Benign	Cancer
Test +	47	1
Test -	9	21

Supplementary Figure S4: Details of the supervised partial least squares-discriminant

analysis model used on the 15-metabolite signature with MetaboAnalyst 5.0

Figure S4A shows an overview of the partial least squares-discriminant analysis (PLS-DA) score plots according to the different components. Regardless of the components that may be involved, the PLS-DA is unable to clearly distinguish benign (***blue triangles***) from malignant samples (***red crosses***). The overall PLS-DA coefficient score of the 15 metabolites is shown in **Figure S4B**. In the right column, the relative concentration of the metabolite is represented in blue when reduced or in red when augmented, in benign (***left column***) or malignant (***right column***) thyroid nodules. The permutation tests conducted showed that the 15-metabolite signature was well-fitted and generalizable, as statistically significant *p*-values were found for the prediction accuracy during training (*p*-value < 5e-04; 0/2000; **Figure S4C**) and for the separation distance B/W (*p*-value = 0.015; <30/2000; **Figure S4D**). **Figure S4E** shows the contingency table of the PLS-DA model, and its graphic representation. A probability of 0 or close to 0 would class a sample in the benign category (***white dots***), while a probability of 1 or close to 1 would class a sample in the malignant one (***grey dots***). The names of the samples that were misclassified by the PLS-DA are provided.

Supplementary Table S1: Demographic characteristics of patients with Bethesda II nodules.

N°	Gender	Age	Thyroidopathy	Other affections	Drugs	Thyroid function	EU-TIRADS	US Size (mm)	Thyroid scan	Histological Diagnosis
**B1**	F	67	No	HNSCC<br>HBP<br>Osteoradionecrosis	Atenolol	Euthyroid	3	23,5		-
**B2**	F	34	No	No	None	Euthyroid	3	28		NNN
**B3**	M	70	No	HNSCC	None	Euthyroid	5	33		-
**B4**	F	34	No	No	None	Euthyroid	3	30		-
**B5**	F	57	No	No	No	Euthyroid	5	11		-
**B6**	F	33	No	Breast cancer	Paclitaxel<br>Filgrastim<br>Aprepitant	Euthyroid	4	33,5		-
**B7**	F	36	Subclinical hyperthyroidism	Spasmophilia<br>Viral meningitis	Progestogen-onyl pill	Hyperthyroidism	4	18	Hot nodule	Adenoma
**B8**	F	51	No	Breast cancer	Letrozole	Euthyroid	4	24		-
**B9**	F	51	No	Breast cancer	Letrozole	Euthyroid	4	21		-
**B10**	F	29	Hashimoto	Obesity	None	Hyperthyroidism	3	37,5	Cold nodule	-
**B11**	F	60	No	Hiatus hernia<br>Peptic ulcer	Omeprazole<br>Bromazepam<br>Alprazolam<br>Quetiapine	Euthyroid	4	18		-
**B12**	F	29	Hyperthyroidism	No	None	Hyperthyroidism	3	32,5	Hot nodule	-
**B13**	M	48	No	No	None	Euthyroid	4	21		-
**B14**	M	48	No	No	None	Euthyroid	4	16		-

**B15**	F	48	No	Primary hyperparathyroidism	None	Euthyroid	3	22,5		-
**B16**	M	58	No	No	No	Euthyroid	4	50		-
**B17**	F	68	No	No	No	Euthyroid	3	23		-
**B18**	F	33	No	Intra-abdominal desmoid tumour	None	Euthyroid	2	26,5		-
**B19**	F	63	Hashimoto	HBP	Nebivolol	Hyperthyroidism	4	21		-
**B20**	F	51	No	Asthma	Salbutamol	Euthyroid	3	28		Adenoma
**B21**	F	63	No	No	None	Euthyroid	5	10		-
**B22**	F	57	No	No	No	Euthyroid	3	23		-
**B23**	F	63	Hashimoto	No	Levothyroxin	Substituted hypothyroidism	3	22		-
**B24**	F	33	No	No	None	Euthyroid	3	32,5		NNN
**B25**	F	54	No	No	No	Hyperthyroidism	3	26,5	Cold nodule	-
**B26**	F	63	No	Parapharyngeal abscess	None	Euthyroid	4	24,5		-

Abbreviations:
B, Benign; F, Female; HBP, High blood pressure; HNSCC, Head & neck squamous cell carcinoma; M, Male; NNN, Non-neoplastic nodule.

Supplementary Table S2: Demographic characteristics of patients with benign Bethesda III-IV nodules.

N°	Gender	Age	Thyroidopathy	Other affections	Drugs	Thyroid function	EU-TIRADS	US Size (mm)	Cytological diagnosis	Histological Diagnosis
**IB1**	M	82	No	BPH<br>heart arrythmia<br>Ischemic cardiopathy	Ezetimibe<br>Tamsulosine<br>Bisoprolol<br>Acetylsalicylate<br>Allopurinol	Euthyroid	5	14	IV	NNN
**IB2**	F	58	No	Barlow's disease<br>Multiple sclerosis<br>Asthma<br>Antiphospholipid antibody syndrome<br>Raynaud's disease	Acetylsalicylate	Euthyroid	4	19,5	III	Adenoma
**IB3**	M	40	No	No	No	Euthyroid	5	17,5	III	Adenoma
**IB4**	M	63	No	Ischemic cardiopathy<br>HBP<br>Hiatus hernia	Bisoprolol<br>Clopidogrel<br>Acetylsalicylate<br>Ramipril<br>Omeprazole<br>Alirocumab	Euthyroid	4	44,5	III	Adenoma
**IB5**	M	52	No	No	No	Euthyroid	4	41	III	NNN
**IB6**	M	62	No	Hiatus hernia	No	Euthyroid	4	19	IV (HCN)	NNN
**IB7**	F	35	No	No	No	Euthyroid	4	20	IV	NNN
**IB8**	F	62	No	No	No	Euthyroid	4	12,5	IV (HCN)	Adenoma
**IB9**	F	62	No	No	No	Euthyroid	4	17,5	IV (HCN)	Adenoma
**IB10**	F	81	No	Pulmonary embolism	Acetylsalicylate<br>Tinzaparine	Euthyroid	5	19	IV	Adenoma

					Alprazolam					
**IB11**	F	81	No	Pulmonary embolism	Acetylsalicylate<br>Tinzaparine<br>Alprazolam	Euthyroid	5	15	IV	Adenoma
**IB12**	F	50	Hashimoto	No	No	Euthyroid	4	25,5	IV (HCN)	Adenoma
**IB13**	F	60	No	HBP	Ezetimibe<br>Atorvastatine<br>Irbesartan<br>Hydrochlorothiazide	Euthyroid	4	28,5	IV	Adenoma
**IB14**	F	76	No	Cardiac arrythmia<br>Obesity	Omeprazole<br>Cholecalciferol	Euthyroid	3	35	IV (HCN)	NNN
**IB15**	M	75	No	Dyslipidemia	Ezetimibe<br>Simvastatine	Euthyroid	5	19,5	IV	NNN
**IB16**	F	58	No	Barlow's disease<br>Multiple sclerosis<br>Asthma<br>Antiphospholipid antibody syndrome<br>Raynaud's disease	Acetylsalicylate	Euthyroid	4	19,5	IV (HCN)	Adenoma
**IB17**	M	76	No	No	No	Euthyroid	5	18	III	NNN
**IB18**	F	76	No	Cardiac arrythmia<br>Obesity	Omeprazole<br>Cholecalciferol	Euthyroid	4	26	III	Adenoma
**IB19**	M	55	Hashimoto	No	No	Euthyroid	3	27	IV	NNN
**IB20**	F	55	No	No	No	Euthyroid	3	20	IV	Adenoma
**IB21**	F	59	Hashimoto	No	Levothyroxin	Substituted hypothyroidism	3	26,5	III	Adenoma
**IB22**	F	64	Hashimoto	No	No	Euthyroid	4	19	IV (HCN)	NNN
**IB23**	F	35	No	No	No	Euthyroid	4	53	IV (HCN)	NNN

**IB24**	F	51	No	Peptic ulcer<br>Asthma<br>Depression	Rabeprazole<br>Escitalopram<br>Terbutaline<br>Beclometasone<br>Formoterol	Euthyroid	4	48	III	NNN
**IB25**	F	79	No	Diabetes mellitus<br>HBP	Gliclazide	Euthyroid	4	13	III	Adenoma
**IB26**	F	33	No	No	Levothyroxin	Substituted hypothyroidism	4	28	IV (HCN)	NNN
**IB27**	F	67	No	Obesity<br>Hypercholesterolemia<br>Asthma<br>Obstructive sleep apnea	Fenofibrate	Euthyroid	3	34	IV	NNN
**IB28**	F	70	No	No	No	Euthyroid	5	9,6	III	NNN
**IB29**	F	59	Hashimoto	No	Levothyroxin	Substituted hypothyroidism	3	26,5	IV	Adenoma
**IB30**	M	66	No	No	No	Euthyroid	4	49,5	IV	Adenoma

Abbreviations:
BPH, Benign prostatic hyperplasia; F, Female; HBP, High blood pressure; IB, Lesions cytologically classified as Bethesda III (atypia of undetermined significance, AUS) or Bethesda IV (FN, Follicular neoplasm; HCN, Hürtle cell neoplasm; sFN, Suspicious for follicular neoplasm) further diagnosed as benign lesions; M, Male; NNN, Non-neoplastic nodule.

Supplementary Table S3: Demographic characteristics of patients with malignant Bethesda III-IV nodules.

N°	Gender	Age	Thyroidopathy	Other affections	Drugs	Thyroid function	EU-TIRADS	US Size (mm)	Cytological diagnosis	Histological Diagnosis	pT	pN
**IM1**	F	36	No	COPD<br>Hiatus hernia	None	Euthyroid	4	13,5	III	PTC Vesicular	1a	0
**IM2**	M	51	No	No	Pregabaline<br>Bromazepam<br>Paracetamol<br>Clonazepam	Euthyroid	4	25	III	PTC Classical	2	1a
**IM3**	M	51	No	No	Pregabaline<br>Bromazepam<br>Paracetamol<br>Clonazepam	Euthyroid	4	23	III	PTC Classical	2	1a
**IM4**	M	66	No	Ischemic cardiopathy<br>BPH<br>HBP	Amlodipine<br>Acetylsalicylate<br>Levothyroxin<br>Ezetimibe<br>Atorvastatine	Substituted hypothyroidism	4	13	IV	PTC Vesicular	1a(3)	0
**IM5**	F	51	No	Asthma	Salbutamol	Euthyroid	5	10,5	III	PTC Classical	1b	1a
**IM6**	F	63	No	No	None	Euthyroid	5	10	III	PTC Classical	1a	1b
**IM7**	F	20	Hashimoto	No	None	Euthyroid	5	8	IV	PTC Vesicular	1a(3)	1a
**IM8**	F	45	No	Lung adenocarcinoma<br>Pituitary adenoma	Cabergoline	Euthyroid	4	18,5	IV (HCN)	PTC Oncocytic	1b	0
**IM9**	F	37	No	Anemia<br>Obesity<br>Peptic ulcer	Folate<br>Ferrous sulphate	Euthyroid	4	38	IV	PTC Vesicular	2	0
**IM10**	M	32	No	Ankylosing spondylarthritis	Adalimumab	Hyperthyroidism	4	39	IV	PTC Vesicular	2	X

**IM11**	F	30	No	Antiphospholipid antibody syndrome	Levothyroxin	Substituted hypothyroidism	5	15	III	PTC	Classical	1b	0

Abbreviations:
BPH, Benign prostatic hyperplasia; COPD, Chronic obstructive pulmonary disease; IM, Lesions cytologically classified as Bethesda III (atypia of undetermined significance, AUS) or Bethesda IV (FN, Follicular neoplasm; HCN, Hürtle cell neoplasm; sFN, Suspicious for follicular neoplasm) further diagnosed as malignant lesions; F, Female; HBP, High blood pressure; M, Male; PTC, Papillary thyroid carcinoma.

<u>Supplementary Table S4:</u> Demographic characteristics of patients with malignant Bethesda V-VI nodules.

N°	Gender	Age	Thyroidopathy	Other affections	Drugs	Thyroid function	EU-TIRADS	US Size (mm)	Cytological diagnosis	Histological Diagnosis		pT	pN
**M1**	M	55	No	No	Amlodipine<br>Insulin<br>Metformine<br>Glibenclamide<br>Irbesartan<br>Hydrochlorothiazide	Euthyroid	5	15,5	VI	PTC	Sclerosant	3a	1a
**M2**	M	55	No	No	Amlodipine<br>Insulin<br>Metformine<br>Glibenclamide<br>Irbesartan<br>Hydrochlorothiazide	Euthyroid	5	7	VI	PTC	Sclerosant	3a	1a
**M3**	F	35	No	No	No	Euthyroid	5	13	V	PTC	Tall cell	1a	1a
**M4**	F	31	No	No	Levothyroxin	Euthyroid	5	28	V	PTC	Classical	2	0
**M5**	F	21	No	No	Doxycycline<br>Estrogen-progestin pill	Euthyroid	5	20	VI	PTC	Classical	1b	1a
**M6**	F	51	No	Breast cancer	No	Euthyroid	5	12,5	VI	PTC	Classical	1b (2)	1b
**M7**	F	75	No	No	No	Euthyroid	5	7,5	V	PTC	Classical	1a	0
**M8**	F	50	Hashimoto	Pituitary adenoma	No	Euthyroid	4	18	V	PTC	Classical	1b	0
**M9**	F	56	No	HBP	Irbesartan	Euthyroid	5	15,5	VI	PTC	Classical	1b	1a
**M10**	F	21	Hashimoto	No	No	Euthyroid	5	21,5	VI	PTC	Classical	1b	1a
**M11**	M	29	No	No	No	Euthyroid	4	11	V	PTC	Solid	2 (2)	0

Abbreviations:

BPH, Benign prostatic hyperplasia; COPD, Chronic obstructive pulmonary disease; ***Mx***, Lesions cytologically classified as Bethesda V (suspicious for malignancy) or Bethesda VI (malignant) and confirmed by histopathology examination; F, Female; HBP, High blood pressure; M, Male; PTC, Papillary thyroid carcinoma.
